# The vulnerable microcirculation in the critically ill pediatric patient

**DOI:** 10.1186/s13054-016-1496-x

**Published:** 2016-10-30

**Authors:** J. W. Kuiper, D. Tibboel, C. Ince

**Affiliations:** 1 Intensive Care and Department of Pediatric Surgery, Erasmus Medical Center – Sophia Children’s Hospital, Postbox 2040, 3000 CA Rotterdam, The Netherlands; 2 Department of Intensive Care, Erasmus MC, University Medical Center Rotterdam, ‘s-Gravendijkwal 230, 3015 CE Rotterdam, The Netherlands

**Keywords:** Microcirculation, Pediatrics, Hemodynamic coherence

## Abstract

In neonates, cardiovascular system development does not stop after the transition from intra-uterine to extra-uterine life and is not limited to the macrocirculation. The microcirculation (MC), which is essential for oxygen, nutrient, and drug delivery to tissues and cells, also develops. Developmental changes in the microcirculatory structure continue to occur during the initial weeks of life in healthy neonates. The physiologic hallmarks of neonates and developing children make them particularly vulnerable during critical illness; however, the cardiovascular monitoring possibilities are limited compared with critically ill adult patients. Therefore, the development of non-invasive methods for monitoring the MC is necessary in pediatric critical care for early identification of impending deterioration and to enable the initiation and titration of therapy to ensure cell survival. To date, the MC may be non-invasively monitored at the bedside using hand-held videomicroscopy, which provides useful information regarding the microcirculation. There is an increasing number of studies on the MC in neonates and pediatric patients; however, additional steps are necessary to transition MC monitoring from bench to bedside. The recently introduced concept of hemodynamic coherence describes the relationship between changes in the MC and macrocirculation. The loss of hemodynamic coherence may result in a depressed MC despite an improvement in the macrocirculation, which represents a condition associated with adverse outcomes. In the pediatric intensive care unit, the concept of hemodynamic coherence may function as a framework to develop microcirculatory measurements towards implementation in daily clinical practice.

## Background

The cardiorespiratory system delivers oxygen and nutrients to meet oxygen and nutrient demands to support cellular and organ function. Hemodynamic monitoring is vital to identify changes in clinical conditions and evaluate interventions. It is important that hemodynamic monitoring is easily applied, reproducible, quantitative, and warns the physician before hemodynamic deterioration leads to cellular and organ injury. In addition to hemodynamic monitoring, a thorough understanding of physiology and pathophysiology is important in the care of critically ill patients. However, the pediatric intensivist and anesthesiologist face specific age- and development-related problems, such as different body proportions, increased metabolic rate, and reduced respiratory and cardiovascular reserves, which makes the care of these patients particularly challenging.

This review focuses on the role of monitoring the microcirculation (MC) in (not yet) hemodynamically unstable pediatric patients and the specific problems that pediatric intensivists and anesthesiologists face on a daily basis in the care of this heterogeneous patient group. Cardiovascular development, the role of oxygen during development in the pediatric population, and the role of the MC are described. The specific problems encountered during hemodynamic instability, the limitations of cardiovascular monitoring, and how monitoring the MC may aid in decision making when initiating or evaluating therapies are subsequently discussed. In this context, we focus on the recently introduced concept of hemodynamic coherence (HC), which may provide a framework for future decision-making. HC describes the relationship between the MC and the macrocirculation. When HC is present, improvements in the macrocirculation will lead to improvements in the MC. However, an optimally functioning macrocirculation does not guarantee an adequate microcirculatory perfusion. In specific situations, such as sepsis, HC is lost and despite an improved macrocirculation, the MC may remain dysfunctional. Furthermore, when attempts to improve the macrocirculation do not improve the MC, additional interventions may worsen the MC [1]. The review concludes with a discussion regarding microcirculatory targeted therapy in the future.

### Cardiovascular development

Cardiovascular development in children is a highly complex process. Major changes with important physiological consequences occur in the initial hours to days following birth. Further growth and development during the neonatal period and infancy change the physiology much less dramatically and the cardiovascular physiology begins to more closely resemble the adult physiology. The physiology in younger children continues to change but the physiology of older children becomes similar to adult physiology. Cardiovascular development affects important physiological parameters, such as the pulmonary and systemic vascular resistance (SVR), ventricular stroke volume, organ blood flow, and heart rate and thus compensatory mechanisms [2].

The transition from fetal to extra-uterine life is a complex process that affects nearly every organ; however, major changes predominately occur in the cardiovascular system. Fetal life is characterized by the presence of the low resistance placental circulation and fetal communication, the ductus arteriosus, the ductus venosus, and the foramen ovale, as well as development in a relatively hypoxic environment with high hematocrit. The left and right ventricular pressures are equal with right heart predominance [2]. In addition, the pulmonary vascular resistance is high and changes during fetal life, whereas a substantial decrease in the vascular resistance occurs after birth [3, 4]. Of note, the pulmonary vascular reactivity to oxygen increases during pregnancy, with potential implications for pulmonary hypertension and right to left shunting after birth [3, 4]. After clamping the umbilical cord, the fetal circulation rapidly transforms into an adult circulation. Under the influence of various hormones released during labor and delivery, as well as the removal of the low resistance placental circulation, the SVR suddenly increases, whereas the pulmonary vascular resistance decreases [2]. These changes result in increased pulmonary blood flow and increased left ventricle preload. The result is an increased left ventricular output that peaks 2 h after birth [5, 6]. Following the closure of the foramen ovale, the ductus arteriosus closes, which partially explains the decrease in the left ventricular output in the 22 h after the peak left ventricular output [2]. The closure of the ductus arteriosus is typically complete after 48–72 h, although it may be delayed when shunting across the ductus persists [6]. The separation of the pulmonary and systemic circulation leads to an arterial oxygen saturation increase from 60–70 % one minute after birth to near normal adult values after 8–10 minutes [5, 7, 8]. The ventricles of newborns are less compliant with decreased diastolic function; moreover, the response to inotropes and volume loading are less pronounced and an increased afterload is less well tolerated [9]. Both the SVR and left ventricular afterload increase; thus, there is limited inotropic reserve.

Following the initial drastic changes in the cardiovascular physiology after birth, the cardiovascular system continues to change more slowly and the differences between child physiology and adult physiology become less clear [10]. In the first few years following birth, the heart adapts to the new preload and afterload and the inotropic reserve capacity increases [9]. After its initial increase, the SVR decreases, particularly in the first 5 years of life. In this same period, the stroke volume index increases and stabilizes to adult values at approximately 5 years of age. The stroke volume continues to increase until 13 years of age. The cardiac index increases in the first 3 years but decreases after 5 years of age to adult levels after 10 years of age [10].

The normal transition and cardiovascular development are well-balanced processes; however, the physiological remnants of fetal life and the consequences of a developing cardiovascular system may become obvious when the normal physiology is under pressure and compensatory mechanisms are needed. In critically ill children in the first years of life, the oxygen and metabolic demands are increased, exhibit less variation, and lack a hypermetabolic response [11]. During sepsis in adults, despite an increased end diastolic volume, septic cardiac dysfunction causes an increased end systolic volume and the stroke volume index remains within the normal range [12]. In addition, the heart rate and cardiac index both increase during sepsis in adults [12, 13]. In children with septic shock, despite impaired contractility, the left ventricular preload presumably does not increase and compensation mainly depends on an increasing heart rate. This is limited by the normally high heart rate in neonates and infants and, to a lesser extent, in children past infancy [14]. However, more recent studies demonstrate that increases in the cardiac output (CO) can be achieved by an increased stroke volume [15–18]. Cardiac function in neonates and infants is characterized by an increased contractile state, increased sensitivity to afterload, and increased oxygen demands at an increased heart rate or preload state, which is less clear for children [15, 16]. In contrast to adults, in which a high cardiac index and low SVR are the hallmarks of sepsis, the presentation of pediatric patients differs. In central venous catheter-related sepsis 94 % of the pediatric patients presented with high cardiac index and low SVR but in community acquired sepsis 86 % presented with a low to normal cardiac index and variable SVR [19].

### Assessment of cardiovascular compromised pediatric patients

The currently available techniques to assess cardiovascular compromised pediatric patients and evaluate therapy include both invasive techniques, such as the direct measurement of CO, and non-invasive techniques, such as echocardiography and physical examination. Furthermore, central and mixed venous saturation have been used as surrogate markers for the adequacy of CO. The capillary refill time, peripheral temperature, and serum lactate levels are used as markers of tissue perfusion, and measurements of the lung water and “fluid responsiveness” may be used to guide fluid therapy [20]. Persistently low CO measurements in septic children are associated with increased mortality [21, 22].

The clinical estimation of CO is unreliable, which has been demonstrated in infants and children following cardiac surgery [23]. The gold standard for invasive measurement of CO is the pulmonary artery catheter, which is not widely used in children [24, 25], not only because of its size but also because pulmonary artery catheter usage in adults has not been demonstrated to be effective and is associated with an increased length of stay, mortality, and costs [26–28]. Less invasive alternatives have been developed, such as Doppler signals, dilution-based methods, and bioimpedance. Following its use for approximately 20 years in pediatrics, Lemson et al. validated transpulmonary thermodilution in lambs as a precise method to measure CO [24, 25, 29]; however, it requires both central venous and arterial access and is typically unsuitable for infants under 3.5 kg. Moreover, the validity and relevance of CO measurement using transpulmonary dilution techniques in patients with intra-cardiac or extra-cardiac shunts is questionable. Echocardiographic estimations of CO are highly operator-dependent, require extensive training, and are, therefore, often inaccurate; moreover, research in children focuses on different techniques and its validation under different conditions [20, 30]. Venous oximetry comprises an invasive measurement; however, it is used as an alternative for invasively measured CO. Central venous saturation represents a poor surrogate for determining the adequacy of CO [31]. Central venous saturation is typically measured in the superior vena cava because measurements from the inferior vena cava may provide deviating results [32, 33]. A further complicating feature in the interpretation of venous saturation is the matter of high mixed venous saturations in critically ill patients as a result of impaired oxygen extraction and cardiac and microcirculatory shunts [9, 20, 34]. Thus, it is unclear whether central venous saturation measurements have additional value in pediatric critical care; nevertheless, central venous saturation is part of the Surviving Sepsis Campaign protocol for children [20, 35]. In summary, CO measurements in children are difficult and are much less frequently performed compared with adults.

For the assessment of tissue perfusion, the capillary refill time, temperature, and serum lactate concentrations are used. A prolonged capillary refill time may indicate an early warning sign of cardiovascular failure. Despite a predictive value in an emergency department [36, 37], in an intensive care unit, the capillary refill time exhibits no correlation with hemodynamic variables, such as the cardiac index, central venous pressure, stroke volume index, and SVR, in pediatric patients following cardiac surgery [38]. In the general pediatric intensive care unit population, only a severely prolonged capillary refill time correlates with the stroke volume index and lactate concentration; however, this correlation is relatively weak [38]. In this same study, Tibby et al. demonstrated that the core-peripheral temperature gap closely correlated with the capillary refill time; however, there was no correlation with the previously described hemodynamic parameters [38]. Lactate is used as a marker of tissue perfusion but increased lactate levels may also arise from increased production via activated white blood cells, inflammatory mediator-accelerated glycolysis and catecholamine-stimulated muscle, or decreased clearance during mitochondrial dysfunction or liver failure [39, 40]. In various circumstances, a high serum lactate concentration has prognostic value in a pediatric intensive care unit [41–45]. To date, however, there is no evidence in pediatric patients indicating improved outcome when a reduction in lactate levels is used as a target in goal-directed therapy [39, 46].

Taken together, no bedside tool to date reliably informs the pediatric intensivist or pediatric anesthesiologist regarding oxygen delivery in critically ill pediatric patients or is able to warn the clinician of impending cardiovascular deterioration. Most therapeutic interventions aim to improve oxygen delivery to the tissues and cells. However, the effect of blood transfusions to optimize the oxygen-carrying capacity of the circulation, fluid administration, inotropes, and vasopressors is measured using the previously described (macrocirculatory) parameters as end points. However, these interventions only improve oxygen delivery to the tissues and cells when HC is preserved, i.e., the effects on the macrocirculatory parameters are also effective in restoring the MC [1]. Also, the effects of these interventions on the MC are not yet clinically monitored at the bedside.

### Monitoring of the pediatric MC

The final steps in oxygen delivery by the cardiovascular system are to deliver oxygen to the MC and diffusion of oxygen to cells. Hand-held videomicroscopy, utilizing orthogonal polarization, spectral, sidestream dark field, and incident dark field imaging techniques, may readily be used to visualize the MC at the bedside. Currently, consensus meetings are being held to determine the optimal method to analyze and obtain functional microcirculatory parameters from the images obtained by hand-held videomicroscopy. Several studies in pediatric patients have addressed the microcirculation in various disease states and different age groups [47–49].

The MC consists of vessels with a diameter smaller than 100 μm: arterioles, capillaries, and venules [50]. An optimally functioning macrocirculation does not guarantee adequate microcirculatory perfusion if therapeutic interventions do not result in a coherent improvement of the MC. Parameters such as the arteriolar tone, hemorheology, endothelial function, and capillary patency also determine flow in the MC [50]. In adult patients with severe sepsis and traumatic hemorrhagic shock, the loss of coherence between the resuscitated macrocirculation and MC has been demonstrated to be the most sensitive and specific hemodynamic indicator associated with increased multi-organ failure and mortality [51–56]. In critically ill children with sepsis, a persistently altered MC has been associated with increased mortality [48]. Indices of microcirculatory blood flow may also serve as early indicators of decreased perfusion of the MC and potentially warn clinicians regarding the development of multi-organ failure [57–59]. Thus, MC measurement must be considered a valuable extension of current hemodynamic monitoring techniques [9, 60].

Readily accessible sites for MC measurements using hand-held videomicroscopy include the buccal and sublingual MC. In (preterm) neonates, the reduced thickness of the skin enables transcutaneous measurements of the MC [61–63]. Orthogonal polarization spectral imaging was the first hand-held videomicroscopy technique that visualized the MC and was succeeded by sidestream dark field imaging in 2007 [64–66]. Recently, the CytoCam, which is based on incident dark field imaging, was introduced [67]. The CytoCam uses a different illumination technique and is smaller and lighter and has a higher optical resolution [68]. In preterm neonates, transcutaneous measurements using an incident dark field have been demonstrated to be superior to a sidestream dark field in terms of the detected number of vessels, and the perfusion of the vessels could also be more accurately detected [68]. Moreover, the imaging quality scores for illumination, focus, and pressure were better for the incident dark field compared with sidestream dark field imaging [68].

To differentiate capillaries from venules, in general, a cutoff value of 20 μm is used [69]. In preterm and term neonates, however, small vessels are, on average, 8.4 μm; moreover, in term neonates, the capillary diameter does not exceed 10 μm [47]. Thus, in pediatric studies, a general cutoff value of 10 μm is used [48, 49, 70–74]. Ideally, multiple measurements should be performed per organ in at least three different sites [69]. To describe the MC, descriptive parameters such as perfused vessel density, as a measure of functional capillary density, have been introduced. The functional capillary density represents the functional volume of flowing red blood cell (RBC)-filled capillaries per unit area of tissue. Additional parameters include the proportion of perfused vessels and total vessel density [69]. The measurement of the flow is the major technical challenge, which is why a semi-quantitative index referred to as the microvascular flow index has been developed [75, 76]. It must be noted that this index was specifically designed for sepsis and may not be well suited for other states of hypoperfusion, such as heart failure with a progressive decrease in flow. Depending on the disease state, the MC may be very heterogeneous, particularly during distributive shock as exhibited in sepsis [59]. To this end, a heterogeneity index was introduced to describe this property of the MC [59, 69]. A recent paper provides a comprehensive review regarding the current state-of-the-art of hand-held videomicroscopy and the difficulties encountered in the application of this technique [77]. Nevertheless, visualization of the MC has been demonstrated to be feasible and has introduced a novel field of research and a new modality for non-invasive hemodynamic monitoring in the pediatric population (Tables 1 and 2).

**Table 1:** Neonatal studies of the microcirculation using orthogonal polarization spectral, sidestream dark field or incident dark field imaging

Study	*N*	Age group	Technique/site	Disease	Intervention	Outcome
Genzel-Boroviczény et al. 2002 [47]	37	Preterm/term	OPS/skin	-	-	Feasibility study; RBC velocity increases in preterm infants, correlated with a decrease in Hb
Genzel-Boroviczény et al. 2004 [61]	13	Preterm	OPS/skin	Anemia	RBC transfusion	FCD improves at least 24 h
Kroth et al. 2008 [62]	25	Preterm	OPS/skin	-	-	FCD decreases in first 4 weeks of life, correlated with Hb levels and incubator temperature
Top et al. 2009 [70]	14	0–18 days	OPS/buccal	Respiratory failure	-	FCD is decreased compared with non-ventilated controls, VA-ECMO improves FCD
Weidlich et al. 2009 [87]	25	Preterm	OPS/skin	Infection	-	FCD decreases 1 day before clinical signs of infection
Hiedl et al. 2010 [81]	25	Preterm	SDF/skin	PDA	PDA closure	Reduces FCD with PDA, recovery of FCD after closure of PDA
Ergenekon et al. 2011 [63]	15	Term	SDF/skin	Polycythemia	PET	PET improves FCD
D’Souza et al. 2011 [111]	115	Preterm/term	OPS/skin	LBW	-	FCD is increased in LBW compared with normal birth weight infants
Top et al. 2012 [73]	21	Term	OPS/buccal	Respiratory failure	VA-ECMO	FCD is maintained but not immediately improved following initiation of VA-ECMO
Ergenekon et al. 2013 [112]	14	Term	SDF/skin	Asphyxia	TH	Flow is impaired compared with controls, flow improves after re-warming
Alba-Alejandre et al. 2013 [113]	47	Term	OPS/skin	Infection		Flow is decreased during infection, no difference in FCD
Schwepcke et al. 2013 [114]	21	Preterm	SDF/skin	Hypotension		FCD is increased in hypotensive neonates 6 h after birth; it subsequently normalized to normotensive control levels
Raghuraman et al. 2013 [115]	141	Preterm/term	OPS/skin	-	-	FCD is increased in twins compared with singletons, low birth weight was associated with lower FCD
Buijs et al. 2014 [74]	56	Term	SDF/buccal	CDH	-	Loss of hemodynamic coherence; severely impaired MC after dopamine predicts need for the addition of (nor)epinephrine
Van den Berg et al. 2014 [116]	28	Term	SDF	-	-	Buccal measurements of vessel density are reproducible, cutaneous are not reproducible
Van Elteren et al. 2015 [68]	20	Preterm	SDF/IDF	-	-	IDF is superior compared with SDF in vessel visualization, visualization of perfusion, and image quality score

*CDH* congenital diaphragmatic hernia, *FCD* functional capillary density, *Hb* hemoglobin, *IDF* incident dark field imaging, *LBW* low birth weight, *OPS* orthogonal polarization spectral imaging, *PDA* persistent ductus arteriosus, *PET* partial exchange transfusion, *RBC* red blood cell, *SDF* sidestream dark field imaging, *TH* therapeutic hypothermia, *VA-ECMO* veno-arterial extracorporeal membrane oxygenation

**Table 2:** Pediatric studies of the microcirculation using orthogonal polarization spectral or sidestream dark field

Study	*N*	Age group	Technique/site	Disease	Intervention	Outcome
Top et al. 2010 [71]	45	0–3 years	OPS/buccal	-	-	FCD decreases after the first week of life
Top et al. 2011 [48]	18	0–15 years	OPS/buccal	Septic shock	-	FCD does not differ on day 1, non-survivors have persistently low FCD
Top et al. 2011 [72]	8	0–3 years	OPS/buccal	Respiratory failure	iNO	iNO increases FCD without altering macrocirculatory parameters
Paize et al. 2012 [117]	60	0–6 years	SDF/sublingual	MCD	-	FCD is decreased at admission; however, it increases when MCD resolves. HI is correlated with duration of ventilation
Buijs et al. 2014 [49]	20	0–16 years	SDF/buccal	Cardiac arrest	TH	FCD and flow are impaired during TH; however, they recover after re-warming. Severe impairment was associated with mortality
Nussbaum et al. 2015 [85]	40	0–3 years	SDF/ear	Cardiac surgery/catheterization	-	Transient reduction in MFI and PVD after cardiac surgery with and without cardiopulmonary bypass
Schinagl et al. 2016 [86]	37	Unknown	SDF/buccal	Anemia	Blood Tx	Transfusion increased TVD with decreased RBC velocity, particularly during infection

*FCD* functional capillary density, *HI* heterogeneity index, *iNO* inhaled nitric oxide, *MCD* meningococcal disease, *MFI* microvascular flow index, *OPS* orthogonal polarization spectral imaging, *PVD* perfused vessel density, *RBC* red blood cell, *SDF* sidestream dark field imaging, *TH* therapeutic hypothermia, *TVD* total vessel density, *Tx* transfusion

### The MC in critically ill pediatric patients

Several studies in pediatric patients have assessed the MC in different clinical settings and disease states. Most studies have been performed in preterm and term neonates in which the physiological differences with the adult population vary most. A smaller selection of studies have been performed in the pediatric intensive care unit, with a very heterogeneous group in terms of age, physiology, and underlying diagnoses.

#### Studies in neonates

The MC changes in the first weeks of life in healthy term and preterm infants. In various disease states and different age groups, changes in the MC following interventions have been described (Table 1). Changes in the MC have been associated with poor outcomes in various disease states. Monitoring the MC was first reported in 2002 in preterm and term neonates during the first 5 days of life [47]. The authors demonstrated that the RBC velocity increased in preterm infants during the first 5 days of life and was correlated with a decrease in hemoglobin levels [47]. In the first weeks of life of term and preterm infants, the MC changes likely because of an adaptation to extra-uterine life. In neonates, the functional capillary density decreases in the first week of life [62, 71]. In preterm infants, the functional capillary density of the skin decreases during the first month, which is correlated with the physiological decrease in hemoglobin levels and environmental incubator temperature [62]. The functional capillary density of both the buccal mucosa in term and the skin in preterm infants decreases in the first week, which suggests that these changes represent adaptation to extra-uterine life rather than disturbed development as a result of premature birth [62, 71]. Early research suggests that the MC of the skin resembles that of adults at the age of 3 months [78]. Whether this is true for all microvascular beds remains elusive. Ambient temperature, an increase in oxygen consumption because of the increased work of breathing and gastrointestinal function, and high levels of fetal hemoglobin in the first months of life may be compensated for by increased macrocirculatory and microcirculatory blood flow [9]. The high functional capillary density in the first week of life may be attributed to increased CO and high hematocrit levels, although autoregulation of the MC may also play a role [9, 79, 80].

In various disease states, changes in the MC have been described following interventions (Table 1). In anemic preterm infants, the administration of a blood transfusion resulted in a parallel increment in the functional capillary density, which lasted at least 24 h [61]. Decreasing hematocrit with a partial exchange transfusion in neonates with polycythemia increased the microvascular flow index for small and larger vessels, which suggests an optimal hematocrit for the maximal functional capillary density and microvascular flow index. In neonates with severe respiratory failure, the functional capillary density was decreased compared with non-ventilated controls; however, it significantly increased as the clinical conditions improved and the patients were removed from extracorporeal membrane oxygenation (ECMO) support [70]. The observed improvement in the functional capillary density was most likely a result of a combination of the overall clinical improvement, administration of vasodilators, and decreased levels of vasopressors [70]. During ECMO for severe respiratory failure, the functional capillary density, microvascular flow index, and heterogeneity index did not improve following the initiation of ECMO, although ECMO appeared to prevent the further deterioration of the MC [73]. Preterm infants with a patent ductus arteriosus (PDA) with a left to right shunt that resulted in decreased peripheral perfusion had a lower functional capillary density compared with preterm infants without a PDA [81]. Interestingly, there appeared to be a shift in the microcirculatory flow to relatively smaller vessels in the PDA group [81]. Correction of the PDA resulted in an improvement of the functional capillary density to control values and the shift to relatively more small vessels disappeared [81]. Dopamine improved the macrocirculation in neonates with CDH; however, it did not hemodynamically coherent improve the MC, and the MC also did not improve after the addition of epinephrine or norepinephrine, whereas these treatments did increase the heart rate and blood pressure [74]. Adrenergic vasopressor treatment may be deleterious for the MC [82], and similar results have been demonstrated in adults [83, 84]. It remains unclear how the MC in critically ill neonatal patients may be optimally improved. To date, only blood transfusion during anemia has been reported to improve the MC in terms of increasing the functional capillary density.

#### Studies in pediatric patients

Most studies have been performed in neonates, but several studies have focused on older children as well (Table 2). In patients ranging from 0–3 years old, Top et al. [71] reported that functional capillary density changes in the first week, after which there was no correlation between age and the functional capillary density. Also in patients ranging from 0–3 years old, cardiac surgery with or without cardiopulmonary bypass resulted in a transient reduction of the microvascular flow index and functional capillary density [85]. Only two studies in children demonstrated changes in the MC after an intervention. In eight patients with hypoxemic respiratory failure, five of whom suffered from congenital diaphragmatic hernia, the buccal functional capillary density significantly improved following the initiation of inhaled nitric oxide (NO) [72]. Inhaled NO did not affect the systemic blood pressure, which makes the mechanism by which NO improves the MC unclear [72]. In anemic hematology or oncology patients, a blood transfusion increased the total vessel density but not to normal values [86]. In pediatric patients, similar to neonates, it remains unclear how to best improve the MC in terms of the functional capillary density.

### Monitoring the MC to guide patient management

The studies in pediatric critically ill patients have mainly been observational in nature, which provides vital information but does not guide the initiation or titration of therapy to improve the MC. One study indicated alterations in the MC that preceded clinical deterioration, and a limited number of studies have demonstrated prognostic value (Table 1). Nevertheless, monitoring the MC may provide valuable information regarding the loss of HC. Four types of alterations in the MC have been identified that underlie the loss of HC. Weidlich et al. [87] performed the first study that demonstrated MC measurements may be used to initiate therapy. This study indicated that the functional capillary density decreased one day before changes in the laboratory parameters and preceded the prescription of antibiotics in preterm infants. In septic pediatric patients, a persistently altered MC was prognostic for mortality and was demonstrated to be superior to the severity of the illness score using the pediatric risk of mortality 2 score in the prediction of mortality [48]. Buijs et al. [49] reported poor outcomes in pediatric patients with early buccal microcirculatory impairment during therapeutic hypothermia following cardiac arrest. Re-warming improved the impaired MC to control values; however, the functional capillary density of the vessels with a diameter of 11–100 μm and the microvascular flow index before the initiation of therapeutic hypothermia were significantly decreased in non-survivors [49]. Coherence between the systemic circulation and the MC is lost when improvements in systemic parameters are not reflected by improvements in the MC [1]. This issue may occur particularly in states of shock, inflammation, reperfusion, and infection, as well as resuscitation damage to normal cellular sensing mechanisms [1, 51–54, 88]. In adults, the loss of coherence predominately occurs in sepsis [52, 53, 56]. Loss of HC also occurs in pediatric patients. In neonates with congenital diaphragmatic hernia, increasing the mean arterial blood pressure with catecholamines did not improve the MC [74].

**Fig. 1:**
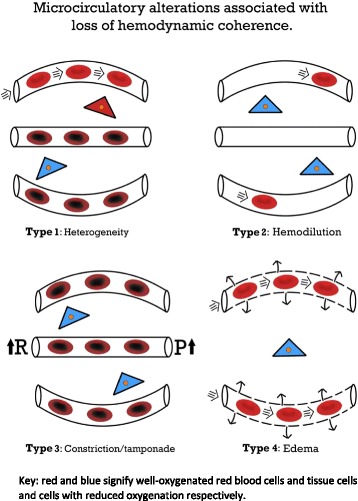
Microcirculatory alterations that underlie the loss of hemodynamic coherence between the macro- and microcirculations resulting in tissue hypoxia. Type 1 comprises a condition with a heterogeneous perfusion of the microcirculation as exhibited by septic patients with obstructed capillaries next to perfused capillaries, which results in a heterogeneous oxygenation of the tissue cells. Type 2 occurs as a consequence of hemodilution, with the dilution of microcirculatory blood resulting in the loss of RBC-filled capillaries and increasing the diffusion distance between oxygen carrying RBCs and tissue cells. Type 3 alterations result in a stasis of microcirculatory RBC flow induced by an increased arterial vascular resistance, vasopressor therapy, increased venous pressure, or hyperoxia. Type 4 alterations involve edema caused by capillary leak syndrome and results in an increased diffusive distance and reduced ability of oxygen to reach tissue cells. Adapted from [1]

Four types of alterations in the MC have been delineated that may underlie the loss of HC (Fig. 1) [1]. The concept of hemodynamic coherence, including the four types that explain the loss of coherence, may become vital when measurements of the MC are ready to be translated into the clinical setting in the future. The four types of loss of coherence have been demonstrated during the previous decade, mainly in adult studies; however, they require further prospective evaluations in pediatric patients, particularly in terms of the effectiveness in improving patient outcome. In type 1, which is typically observed in sepsis, there is heterogeneity in the perfusion of the MC, with obstructed capillaries next to capillaries with normal flow (Fig. 1). The persistence of this type of loss of coherence is associated with a poor outcome in adult sepsis [51, 56]. In pediatric patients who require ECMO, type 1 alterations have been described, which were associated with poor outcome [48]. The heterogeneity index has been introduced to describe type 1 alterations [53]. Type 1 abnormalities warrant the administration of antibiotics and vasoactive agents (vasodilators) to promote the patency of the MC [1, 35], but prospective studies are needed. Type 2 is characterized by hemodilution, which is mainly caused by excessive fluid administration and results in the loss of RBC perfused vessels (Fig. 1). This type has predominately been described in patients who underwent cardiac surgery [89]. This issue may be corrected by blood transfusions to improve the hematocrit levels, which thereby increase the oxygen transport capacity of the microcirculation [90, 91]. Several studies have shown the ability of blood transfusions to improve the MC. Yuruk et al. [90] showed an increase in functional capillary density after blood transfusion, increasing hemoglobin levels from 7.1 to 8.5 g/dL, during cardiac surgery. Comparing transfusions with leukodepleted and non-leukodepleted RBCs in septic patients, Donati et al. [91] showed that increasing hemoglobin levels from 8.3 to 10.4 g/dL with leukodepleted blood improved functional capillary density. This study also suggests that the quality of the transfused blood influences the increase in FCD. Recently, Schinagl et al. [86] showed in pediatric patients with hematological or oncological disease that RBC transfusion, increasing hemoglobin from 7.2 to 8.0 g/dL, improved functional capillary density. Although increasing blood hemoglobin levels may improve the microcirculation in various clinical settings, we do not suggest to change transfusion guidelines. The potential role of inhaled NO in RBC-induced capillary recruitment is unclear. Top et al. [72] showed an improvement of the microcirculation after inhaled NO in pediatric patients with hypoxic respiratory failure. In contrast, Trzeciak et al. [92] did not show improvement of the MC in patients with sepsis following inhaled NO. The effect of RBC transfusion on the MC is likely caused by increasing blood viscosity. In an animal model of anemia it has been shown that increasing the blood viscosity increases functional capillary density (FCD) [93]. The third type comprises the constriction/tamponade type, in which the flow in the MC is constricted (Fig. 1). Norepinephrine, which is advised for use in the treatment of sepsis [35], increases the blood pressure by vasoconstriction, although it simultaneously impairs the RBC flow in the MC [83, 84]. Inappropriate use of other vasopressors has also resulted in this effect [94]. Importantly, hyperoxia is also associated with type 3 alterations of the MC. In healthy volunteers, increasing the fraction of inspired oxygen decreases the sublingual functional capillary density [95]. In animal experiments, administration of hyperbaric oxygen decreased FCD and may be associated with what gives the appearance of maldistribution of perfusion in the MC [96]. Central venous pressures that exceed 12 mmHg may reduce the perfusion of the MC by inducing tamponade, which likely occurs via an increase in the post-capillary pressure [97]. The final type occurs when a capillary leak, endothelial damage, and loss of glycocalyx barriers lead to edema formation with an increased diffusion distance (Fig. 1) [1]. This type may have been involved in patients with severe malaria treated with a liberal fluid strategy [98]. In a trial of pediatric patients with malaria or sepsis, this type of loss of coherence may partially explain the adverse outcome in the liberal fluid administration group [99, 100].

An altered MC may be the cause of injury or a marker of cellular injury, since various insults may cause MC dysfunction. Macrocirculatory dysfunction negatively affects the MC and local inflammation and hormonal actions also affect it [1, 101]. In sepsis, endothelial cell injury and RBC injury may cause dysfunction of the MC. In the absence of injury, however, (iatrogenic) dysfunction of the MC can also occur, for example, following hemodilution or nor-epinephrine infusion [1, 83, 84, 101]. Of special interest is the interaction between the MC and mitochondrial function, particularly during states of shock, sepsis, and resuscitation [102]. When mitochondrial dysfunction occurs, cells may fail to utilize oxygen regardless of the state of the MC [103]. Irrespective of the cause of dysfunction of the MC, during an effective resuscitation of the macrocirculation, the MC should be simultaneously monitored to assure HC. When HC is lost, monitoring the MC and establishing the type of loss of HC can guide therapy. The concept of HC and the four types of alterations that may explain the loss of coherence are based on experience with monitoring the MC in the previous decade; however, this concept requires further prospective evaluations in children and adults. Larger studies, including observational studies and randomized trials, are needed to assess the effectiveness and patient outcome of interventions based on this concept.

### Microcirculatory-targeted therapy in the future

Before measurement of the MC can become a part of the routine care of critically ill children, three central issues need to be addressed. First, larger observational studies are needed to further delineate the role of the MC in various disease states in critically ill children and observe the effect of interventions and outcome. International observational cohort studies have been performed in critically ill adults [104]. In randomized trials the effect of monitoring the MC and intervening in the MC on outcome should subsequently be examined. The second issue is the validation in observational studies and randomized trials of the concept of HC as a framework for bedside monitoring and decision-making in children and adults. Third, future research should focus on the translation from bench to bedside of monitoring the MC. Important research areas are, first, the determination of which functional parameters best describe the physiological (dys)function of the MC. Second, the image quality and analyses should be of high quality and automated to be instantaneously available at the bedside. Third, the establishment of the concept of HC as a framework for MC-targeted therapy at the bedside will require a hardware and software platform that integrates the monitoring of the macrocirculation with the MC.

Physiological and quantitative parameters based on the oxygen transport capacity of the MC will need to be introduced based on the classic MC literature related to oxygen transport to tissues. It is essential for the optimization of the oxygen carrying capacity of the MC to ensure proper convective flow, short diffusion distances, and a sufficient level of hematocrit [105, 106]. For a complete functional description of MC images, four physiological parameters are needed. First, the available RBCs in the capillaries are defined by the *tube hematocrit*, which is measured from the volume of RBCs in the capillaries divided by the volume of the capillary [105, 106]. Second, the oxygen delivery capacity is defined by the discharge hematocrit, which is the tube hematocrit that flows through the capillary per unit of time [105, 106]. Third, the oxygen-releasing capacity of the MC to the tissues is necessary. This is described by the diffusive capacity of the MC (functional capillary density). Capillary blood flow is not equal in all capillaries and all capillaries are not necessarily filled by oxygen-carrying RBCs. This has been described by the heterogeneity index [53]. This microcirculatory variable may be more accurately defined by a quantitative assessment of the flow pattern distributions in the MC. With the current generation of hardware using computer-controlled imaging sensors, the measurements of these four parameters at the bedside are within reach.

These functional parameters will be of use to describe the type of loss of HC present at the bedside (Table 3) and initiate and titrate therapy. From current knowledge, a low discharge hematocrit associated with a low convective flow is indicative of hypovolemia and has been demonstrated to be effectively treated by fluid therapy. However, if the microcirculatory flow is normal or high, irrespective of the presence of clinical surrogates of hypovolemia, such as lactate and oliguria, fluid therapy has been demonstrated to be ineffective in improving the microcirculatory perfusion [107, 108]. Using the optimization of the microcirculatory flow to achieve maximum oxygen transport to tissues as an end point, it has been proposed to administer fluids only following the indication of a low discharge hematocrit as the ultimate definition of hypovolemia. However, when the functional capillary density decreases, fluid administration should be terminated to avoid a type 2 loss of HC [1]. Similarly, microcirculatory-guided therapy has been proposed for the treatment of hypotension by vasopressor therapy. It has been demonstrated that only a low functional capillary density associated with hypotension responded to vasopressor therapy in the presence of a normal or high functional capillary density, despite the finding that the presence of hypotension vasopressor therapy did not improve the microcirculatory flow [83, 84]. These are examples of microcirculatory-guided fluid therapy that may be applicable to pediatric patients, although they must be validated in critically ill pediatric patients first.

**Table 3:** Presumed microcirculatory changes identified via hand-held videomicroscopy for the various types of hemodynamic coherence loss

	Type 1	Type 2	Type 3	Type 4
FCD	↓	↓	↓	↓↓↓
Tube Ht	=	↓↓↓	=/↑	=
Discharge Ht	↓↑	↓↓	↓↓↓	=
HI	↑↑↑	=	=	=

Type 1, flow heterogeneity; type 2, hemodilution; type 3, constriction tamponade; type 4, edema. See text for further information

*FCD* functional capillary density, *Ht* hematocrit, *HI* heterogeneity index

Information from the MC must be integrated with systemic hemodynamic monitoring to provide an integrative hemodynamic monitoring platform of the cardiovascular system to facilitate clinical decisions and the titration of therapies. More sensitive information may also be obtained by further development of hand-held videomicroscopy, such as a prolonged observation of a single microcirculatory unit, inclusion of extra wavelengths of light to measure the RBC oxygen saturation, and an increased magnification to visualize subcellular structures, such as the glycocalyx and tissue membrane junctions [89, 109, 110]. It is expected that with these developments, hand-held videomicroscopy will have substantial benefits, including an early, sensitive diagnosis of cardiovascular compromise and an optimization of therapeutic interventions that restore the function of the vulnerable MC in critically ill pediatric patients.

## Abbreviations

CO: Cardiac output
ECMO: Extracorporeal membrane oxygenation
FCD: Functional capillary density
HC: Hemodynamic coherence
MC: Microcirculation
NO: Nitric oxide
PDA: Patent ductus arteriosus
RBC: Red blood cell
SVR: Systemic vascular resistance
